# Computational Analysis Reveals Distinctive Interaction of miRNAs with Target Genes in the Pathogenesis of Chronic Kidney Disease

**DOI:** 10.3390/genes14040898

**Published:** 2023-04-12

**Authors:** Hafiz Muhammad Umar Salim, Abdullahi Dandare, Fareeha Khalil, Afrose Liaquat, Muhammad Jawad Khan, Aisha Naeem

**Affiliations:** 1 Department of Biosciences, COMSATS University Islamabad, Islamabad 45550, Pakistan; 2 Department of Biochemistry, Usmanu Danfodiyo University, Sokoto P.M.B. 2346, Nigeria; 3 Shifa International Hospital, Shifa Tameer-E-Millat University, Islamabad 45550, Pakistan; 4 Department of Biochemistry, Shifa College of Medicine, Shifa Tameer-E-Millat University, Islamabad 45550, Pakistan; 5 Health Research Governance Department, Ministry of Public Health, Doha P.O. Box 42, Qatar; 6 Department of Oncology, Lombardi Comprehensive Cancer Center, Georgetown University Medical Center, Washington, DC 20057, USA

**Keywords:** biomarker, chronic kidney disease, gene expression, miRNA, pathogenesis

## Abstract

The regulation of genes is crucial for maintaining a healthy intracellular environment, and any dysregulation of gene expression leads to several pathological complications. It is known that many diseases, including kidney diseases, are regulated by miRNAs. However, the data on the use of miRNAs as biomarkers for the diagnosis and treatment of chronic kidney disease (CKD) are not conclusive. The purpose of this study was to elucidate the potential of miRNAs as an efficient biomarker for the detection and treatment of CKD at its early stages. Gene expression profiling data were acquired from the Gene Expression Omnibus (GEO) and differentially expressed genes (DEGs) were identified. miRNAs directly associated with CKD were obtained from an extensive literature search. Network illustration of miRNAs and their projected target differentially expressed genes (tDEGs) was accomplished, followed by functional enrichment analysis. hsa-miR-1-3p, hsa-miR-206, hsa-miR-494 and hsa-miR-577 exhibited a strong association with CKD through the regulation of genes involved in signal transduction, cell proliferation, the regulation of transcription and apoptotic process. All these miRNAs have shown significant contributions to the inflammatory response and the processes which eventually lead to the pathogenesis of CKD. The in silico approach used in this research represents a comprehensive analysis of identified miRNAs and their target genes for the identification of molecular markers of disease processes. The outcomes of the study recommend further efforts for developing miRNA biomarkers set for the early diagnosis of CKD.

## 1. Introduction

Chronic kidney disease (CKD) is a medical situation in which aberrations either in kidney structure or function result in the progressive deterioration of kidney function. It is described by a reduced estimated glomerular filtration rate (eGFR) of less than 60 mL per minute or the presence of other biomarkers of kidney damage for more than 3 months [1]. CKD is characterized by the decline of renal parenchyma and the loss of functional nephrons [2]. The loss of functioning nephrons sets off molecular and cellular processes that cause the remaining nephrons to develop malfunctioning more quickly in compensation [3]. The situation becomes worse as a result of these dysregulated pathways in kidney abnormalities [4]. CKD is a disease of several patho-physiological progressions that result in an anomalous renal function and ultimately kidney damage. It is a rapidly increasing health problem in developing as well as developed countries that leads to a high number of deaths. Among the most serious health consequences of CKD are the development of cardiovascular disease (CVD) and the progression of CKD to end-stage kidney disease (ESKD), which necessitates the use of renal replacement therapy (RRT), e.g., dialysis or renal transplant. The management of CVD and treatment approaches for ESKD impose a significant financial burden on both the affected person and the government. The economic and social impacts associated with CKD constitute a major reason for the increased risk of developing CKD and its progression to a more detrimental stage, e.g., ESKD [5]. A decline in filtration capacity results in the accumulation of injurious metabolites in the blood, posing a serious threat to the individual’s health [6]. On the basis of eGFR, CKD is categorized into five stages of increasing severity [7,8]. The diagnosis of CKD is mainly determined by biomarkers that evaluate kidney function. A comprehensive meta-analysis, encompassing more than 1.5 million subjects, was recently conducted. It was proven that over the span of two years, there was a 30% reduction in the eGFR of subjects anticipating end-stage renal disease (ESRD) and death [9].

CKD symptomatically expresses over years after an extensive dormant period during which the disease remains clinically silent. The metabolic activity of the kidneys is high, and they require a large amount of oxygen. Interstitial capillaries become more permeable in early CKD trauma, allowing many plasma proteins to generate an inflammatory response. A gradual loss in the area of interstitial capillaries progresses to hypoxia within the kidney and interrupts the cell activity involved in the breakdown of collagen, which is synthesized in healthy kidneys. The chronically damaged kidney accumulates collagens, proteoglycans, basement membrane proteins and glycoproteins; the affected region of the fibrotic interstitium is directly related to both renal function and the long-term prognosis of the kidney [10].

The global prevalence of CKD is more than 10%. CKD has evolved as a member of major mortality-causing non-communicable diseases for the past 20 years [11]. A large geographical disparity in the prevalence of CKD has been reported internationally. As an illustration, among the general population of white adults in the United States, the adjusted frequency of CKD stages 3 to 5 ranged from 4.3% in the states of Delaware and Pennsylvania to 16.7% in Florida. It varied from 6.7% in California to 13.4% in the Mid-Western states among African Americans [12]. In all European nations, the same variability has been noted [13]. The adjusted percentage of CKD stages 1 to 5 varied greatly among Asian nations as well, for instance, from 6.7% in southern China to 18.3% in southwest China [14]. The possible reasons for this variability include the sampling method, which may have been misleading, resulting in a sample volume that is not a true representation of the population; demographic differences, e.g., in response rates and ethnicity; variations in laboratory techniques for measurement; and finally, the type of equation used to calculate the eGFR [15].

CKD is often more prevalent with older age and is more prominent in high-income societies among those who are obese, diabetic and hypertensive [16]. In developed countries such as the United States of America, one in three CKD patients also has diabetes and one in five patients has hypertension. These risk factors contribute even more to recently documented cases, i.e., 44% and 29% for diabetes and high blood pressure, respectively [17]. Economic hardship and high medical costs are thought to have implications for people living in underdeveloped nations. Instead of speculating or making assumptions based on Western statistics, it is crucial for developing nations to comprehend the genuine prevalence and nature of the disease [18]. Owing to long silent periods and meager diagnostic options, CKD is diagnosed at mature stages, leaving patients with no other routes except kidney transplant and dialysis [19].

Liang et al. [20] reported that miRNAs constitute new potential biomarkers with an encouraging ability to spot kidney damage at primary stages. These are promising biomarkers for the stage or severity of illness. miRNA is an RNA molecule which is approximately 18–25 nucleotides long but non-coding. It is synthesized from endogenous hairpin-like structured transcripts in the genome [21]. Approximately one thousand miRNAs are encrypted by the human genome, with the potential to regulate approximately 60% genes of the whole genome as they are attached to the coding mRNA because of partial complementarity [22]. Several diseases, including kidney disease, are regulated by miRNAs [20]. miRNAs play an imperative role in the start and advancement of CKD. Levels of miRNAs change as a function of disease development because the discrepancies in miRNA intensities are correlated with the severity of disease [23]. The aim of our study was to shortlist a number of miRNAs which are significantly involved in the onset and progression of CKD which eventually lead to further complications. They may serve as potential diagnostic biomarkers and may also provide effective therapeutic options for better treatment choices at early stages in the future.

## 2. Materials and Methods

After extensive literature mining of scientific search engines such as PubMed and NCBI, relevant studies were shortlisted with a number of related miRNAs differentially expressed in CKD. Studies on *Homo sapiens* miRNAs using microarray in CKD having healthy controls and diseased groups were included. Three studies, namely GSE51674 [24], GSE80247 [25] and GSE89699 [26] (Supplementary Table S1), met our inclusion criteria and we extracted common miRNAs using a Venn diagram. The miRNAs present in at least two experiments were selected for further analysis. Every miRNA was individually searched for its involvement in CKD through online search engines. miRNAs with significant contributions to the disease were included, along with a few non-significant miRNAs to negate or validate the findings. Out of 42, only 12 miRNAs met the inclusion criteria for further analysis.

Four sets of experiments on CKD gene expression profiling by array were identified, namely GSE37171 [27], GSE43484 [28], GSE66494 [29] and GSE142153 [30] (Supplementary Table S2). The methodology followed for the analysis is already reported in detail [31,32,33,34]. Briefly, unprocessed data of these experiments were acquired from Gene Expression Omnibus (GEO) of NCBI, and analyzed using GEO2R to find differentially expressed genes (DEGs) and the level of their expression. The statistical significance was set at *p* < 0.05. GraphPad Prism software version 8.0 (San Diego, CA USA, www.graphpad.com) was used to construct volcano plots for each of the selected experiments. The bioinformatics and evolutionary genomics tool was employed to obtain a shared set of DEGs among the four selected experiments, considered as common DEGs (cDEGs) (list A). The expression pattern of cDEGs was illustrated as a heat map, generated by GraphPad Prism software (version 8).

**Figure 1:**
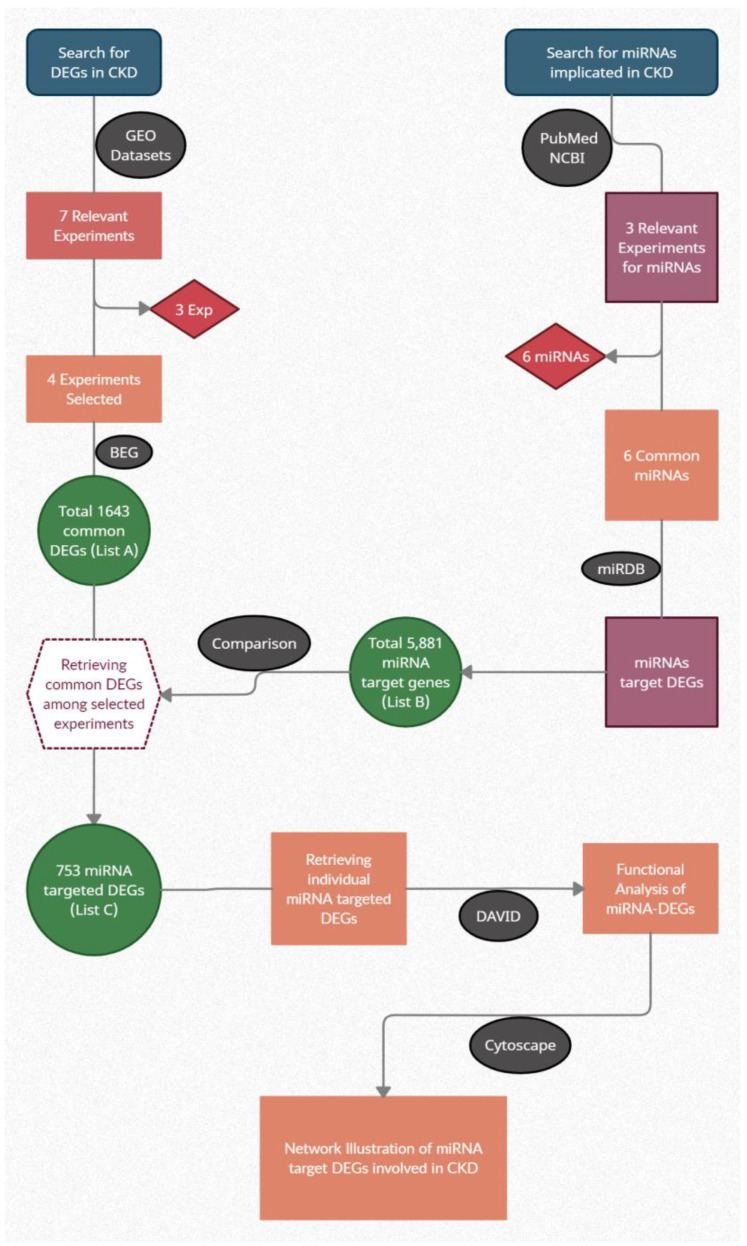
Sequence of work for data extraction and approaches for downstream analysis. CKD: chronic kidney disease, GEO: Gene Expression Omnibus, DB: database, DEGs: differentially expressed genes, miRNA: microRNA, BEG: bioinformatics and evolutionary genomics, DAVID: Database for Annotation, Visualization, and Integrated Discovery.

The potential targeted differentially expressed genes (tDEGs) were acquired from the versatile miRNA databank that is accessible at http://mirdb.org/ontology.html (accessed on 25 May 2022) for each of the 12 included miRNAs. miRNAs with >900 predicted target genes were screened for further analysis. A total of 3955 miRNA target genes were obtained for the 6 miRNAs and retained in Excel format (list B). For the extraction of genes included on both lists A and B, the online platform “Bioinformatics and Evolutionary Genomics” was used. Genes common to list A and list B were considered as miRNA-tDEGs (list C). This list included 456 genes for downstream analysis (Figure 1).

A functional annotation tool, the Database for Annotation, Visualization, and Integrated Discovery (DAVID), version 6.8 (Laboratory of Human Retrovirology and Immunoinformatics, Frederick, MD USA), was employed for functional enrichment analysis of individual lists of miRNA tDEGs. The DAVID online tool helps in providing a comprehensive biological meaning of a set of genes, thereby linking the individual miRNAs with biological processes and molecular functions relevant to the development, worsening or progression of CKD. The list of tDEGs for every independent miRNA was submitted separately to DAVID for processing. The background was set to *Homo sapiens*. The instrument was set to communicate between Gene Ontology term “GO” and every gene identifier primarily in “molecular functions, cellular compartments and biological processes”. For annotation of the pathways for miRNA tDEGs, the Kyoto Encyclopedia of Genes and Genomes (KEGG) was used. For the formation of network relations between miRNAs and their tDEGs involved exclusively in biological processes and in pathways associated with CKD, Cytoscape, version 3.9.0 (Institute for Systems Biology, Seattle, WA USA) was used. The expressions of overlapping genes among the selected experiments were illustrated as heat maps, generated by GraphPad Prism software (version 8).

## 3. Results

**Figure 2:**
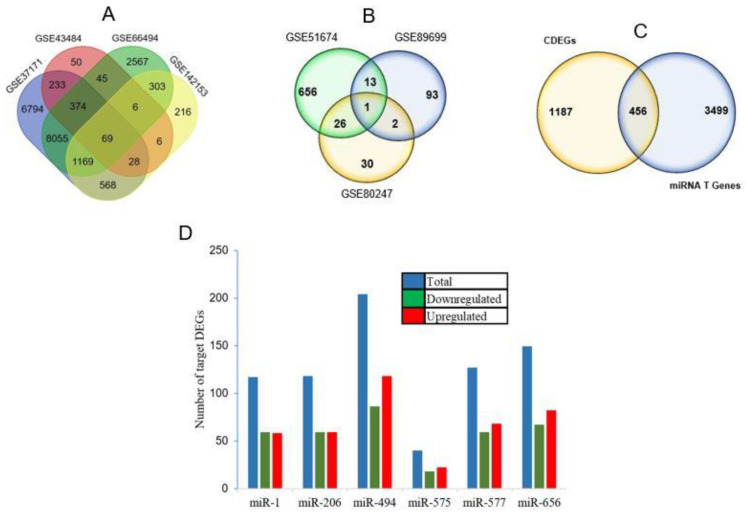
Venn diagram showing (**A**) differentially expressed genes in chronic kidney disease; (**B**) commonly expressed miRNAs in relevant studies; (**C**) targeted differentially expressed genes of all included miRNAs; (**D**) number of target DEGs for individual miRNAs and the number of downregulated and upregulated genes.

Dysregulated genes among four selected experiments are presented in Figure 2A. A total of 1643 DEGs common among the four experiments were established. Only the cDEGs were considered for downstream analyses in order to minimize the probabilities of imprecision. On the other hand, a number of miRNAs among CKD patients reported in different case studies are presented in Figure 2B. There was only one miRNA which was shared by three studies, i.e., hsa-miR-575. Consequently, the miRNAs which were shared by at least two studies were selected for further scrutiny. Six miRNAs were selected from the three studies based on the number of target genes (Table 1) and subsequently the involvement in a greater number of biological processes.

It was observed that 456 cDEGs were potential targets of at least one miRNA and thus considered as miR-tDEGs (Figure 2C). The number of up- and downregulated target DEGs for individual miRNAs is presented in Figure 2D. Each of the six miRNAs has been predicted to control more than 100 genes. miR-494 regulated the maximum number of genes, 204, among which 118 were upregulated and 86 were downregulated. However, miR-575 regulated the lowest number of DEGs, 40 genes, with 22 and 18 genes upregulated and downregulated, respectively.

**Figure 3:**
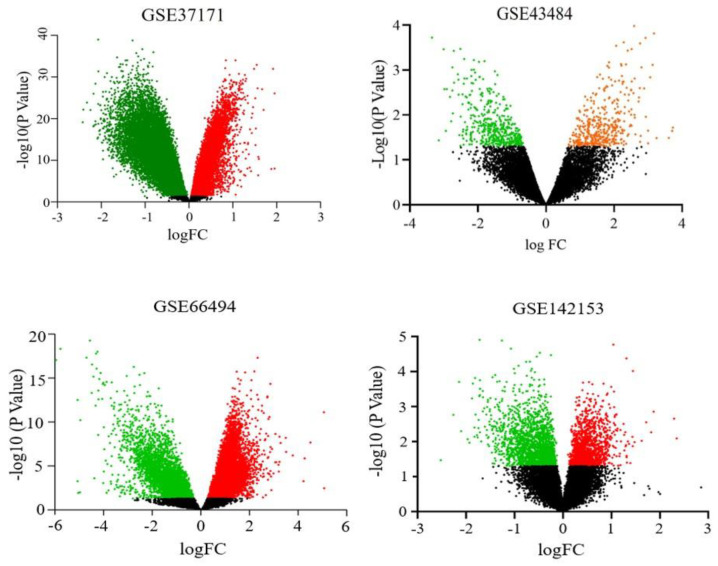
Volcano plots showing the extent of scattering upregulated and downregulated genes for the selected experiments (GSE37171, GSE43484, GSE66494 and GSE142153).

**Figure 4:**
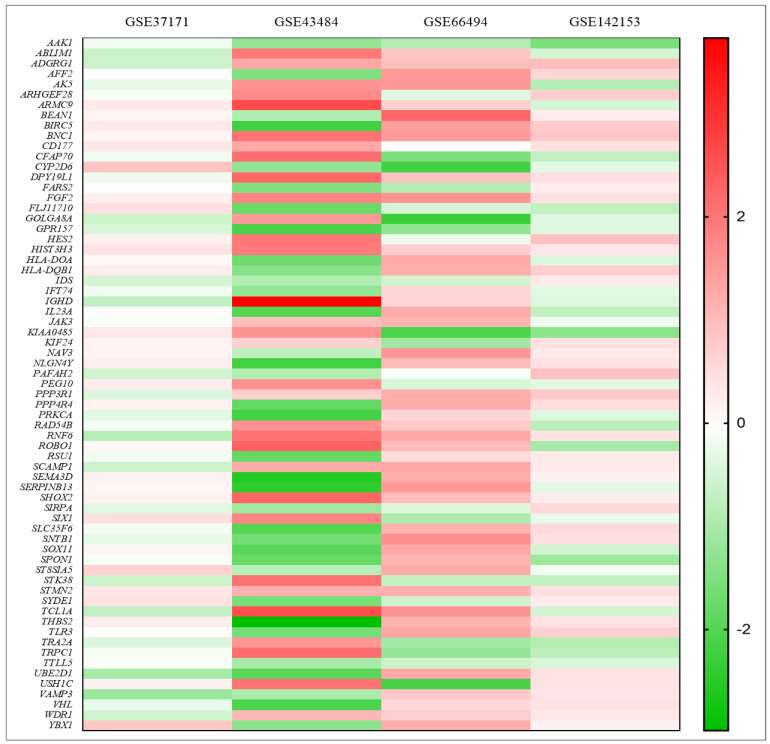
Heat map showing the common differentially expressed genes involved in CKD in four selected studies. Red bands show upregulated genes and green bands indicate downregulated genes.

For a quick visualization of the extent of scattering of both upregulated and downregulated genes, a volcano plot for each selected experiment is presented (Figure 3). Relatively, a large number of DEGs scattered between the fold change of −3 to 3 in the experiments GSE37171 and GSE142153 and between −4 and 4 in GSE43484. However, DEGs of GSE66494 were distributed between −6 and 6 fold changes. Furthermore, the heat map to visualize the variation in gene expression among the selected experiments indicated that most cDEGs maintained the same pattern of expression (i.e., upregulation or downregulation) with varying degrees of fold change, evident from the color intensity (Figure 4).

**Figure 5:**
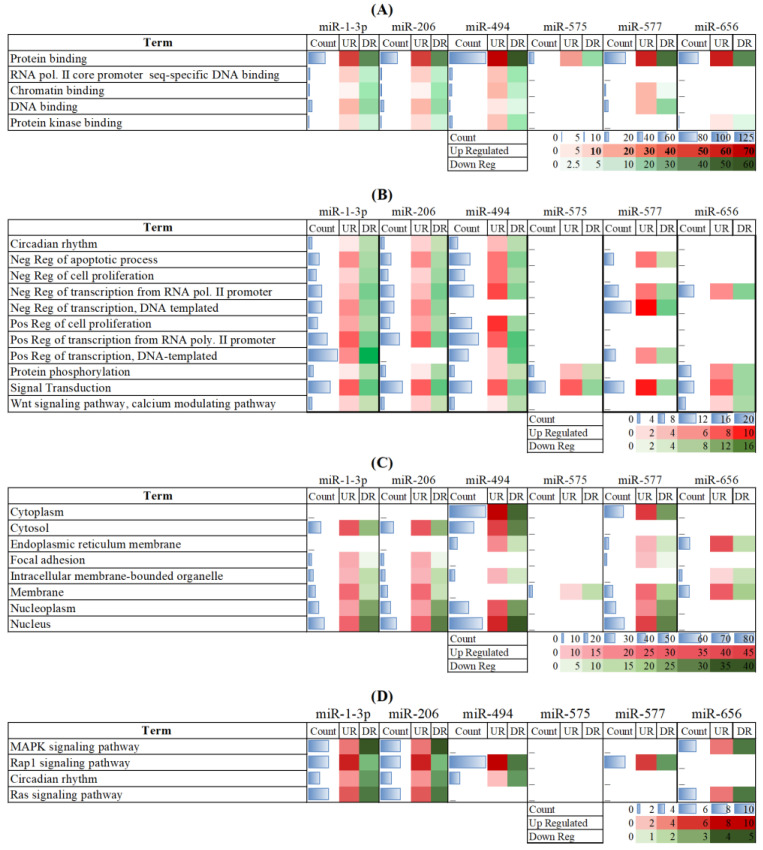
Functional enrichment analysis of miRNA-DEGs. Color intensities show the variation in expression. UR: upregulated genes, DR: downregulated genes. (**A**) Molecular functions. (**B**) Biological processes. (**C**) Cellular compartments. (**D**) KEGG pathways.

The results of Gene Ontology (GO) of miR-tDEGs are presented as molecular functions (MFs), biological processes (BPs), cellular compartments (CCs) and KEGG pathways (Figure 5). miR-494, miR-206 and miR-1-3p exhibited higher levels of contribution to BPs and MFs. This indicated that a comparatively large number of genes regulated by these miRNAs are implicated in CKD. Our results have shown the substantial role of miRNAs in protein binding (Figure 5A), covering a higher number of genes compared to any other MF. All miRNAs took part in regulating genes, particularly in biological roles in membranes (Figure 5C). However, the highest number of genes were expressed inside the nucleus. Moreover, all miRNAs played roles in regulating genes in the nucleoplasm, except miR-656 and miR-575. Conversely, only miR-494 and miR-577 have roles in the cytoplasm.

It was shown that more than one miRNA was predicted to regulate the genes for every GO term without any exceptions. All miRNAs were shown to regulate the gene expression in signal transduction; however, the circadian rhythm was controlled by miR-1-3p, miR-206 and miR-494 through their respective target genes. It is inferred that all prominent MFs and important BPs imperative in CKD were organized by no less than three miRNAs (Figure 5B). The network illustration of the interactions shows that miRNAs and their tDEGs played vital roles in BPs, directly related to CKD. It displays the contribution of individual miRNAs in gene expression regulation in listed BPs.

The results of KEGG pathway analysis are shown in Figure 5D. The pathways in the development or progression of CKD were regulated by miRNA-tDEGs. The following four pathways are shown in the figure: the MAPK signaling pathway, the Rap1 signaling pathway, circadian rhythm and the Ras signaling pathway.

**Figure 6:**
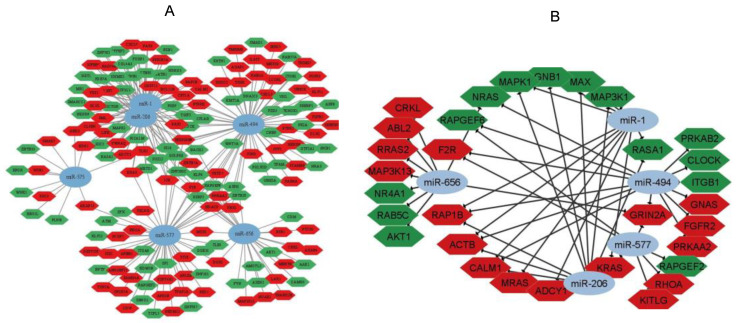
(**A**) Network illustration of miRNAs and tDEGs according to the results of biological process enrichment analysis linked to CKD. (**B**) Network illustration for KEGG pathways associated with miRNA-DEGs.

Figure 6 explains the connections among miRNAs and their target genes in four existing signaling pathways: the MAPK signaling pathway, the Rap1 signaling pathway, circadian rhythm and the Ras signaling pathway. hsa-miR-1-3p and hsa-miR-206 demonstrated the highest involvement in the control of the above-mentioned pathways via the regulation of *GNB1*, *MAPK1*, *ADCY1*, *CALM1*, *RAP1B*, *MAP3K1*, *MRAS*, *MAX*, *RASA1*, *KRAS* and *ACTB* gene expression. miR-575 and miR-577 had the least participation in these signaling pathways, as they are predicted to affect the expression of zero and seven genes, respectively. Every gene was regulated by at least three non-coding RNAs.

## 4. Discussion

Recent studies have suggested the contribution of miRNAs to the regulation of several biological pathways strongly associated with the development of CKD [35]. The purpose of conducting this study was to understand the role of miRNAs and their target genes in the development of CKD to use them as diagnostic or therapeutic markers of CKD. Six miRNAs had robust connections with the disease predicted by the present research. miR-494 had the maximum contribution, regulating 66 genes, and regulates a few pivotal biological processes in the pathogenesis. Moreover, miR-1-3p and miR-206 regulate a combined total of 114 genes. These genes were controlled by more than one miRNA, with only a few exceptions, and all of them were implicated in CKD [27,28,29]. miRNA-494 and miRNA-206 have already been reported to perform key roles in kidney disease [26,36]. miRNA-206 has a role in cell–cell interaction, inflammatory response, intra-cellular signaling and apoptosis [35], whereas miRNA-494 plays protective or injurious roles during acute kidney injury [36].

A number of genes controlled by the analyzed miRNAs were significantly involved in certain biological processes linked to CKD. These include circadian rhythm, negative regulation of apoptotic process, signal transduction, negative regulation of cell proliferation, protein phosphorylation, negative regulation of transcription from RNA polymerase II promoter, negative regulation of transcription, DNA template, positive regulation of cell proliferation, positive regulation of transcription from RNA polymerase II promoter, positive regulation of transcription, DNA-templated synthesis, the Wnt signaling pathway and calcium-modulating pathways. In kidneys, the glomerular filtration rate, renal plasma flow and tubular reabsorption processes are affected by the circadian rhythm. Shifts in the circadian rhythm are associated with the development of CKD and kidney stones [37]. The regulation of circadian rhythm genes by miR-1-3p, miR-206 and miR-494 in the present study showed that the miRNAs play a significant role in the development of CKD. The dysregulated apoptotic process contributes to the pathogenicity of acute kidney injury, i.e., incomplete kidney repair [38]. Over 50% of proteins in human cells are capable of undergoing reversible phosphorylation. Therefore, phosphorylation pathways exhibit promising potential applications in the treatment of a variety of different diseases [39]. Inflammation is an important biological phenomenon in the pathogenesis of CKD. Protein phosphatase has been identified as an effective regulator of a variety of several inflammatory signaling pathways, comprising phosphorylation and de-phosphorylation [40].

A cluster of genes regulated by miR-1-3p participates considerably in the regulation of transcription. Epigenetic and transcriptional profiling through multiple models of chronic kidney damage yield significant comprehensions in the pathophysiological complexity of this intricate disorder. It was found that amplified transcription and gene upregulation were induced due to injury; however, more predominant drivers of gene downregulation are post-transcriptional processes [41]. Transcription factors (e.g., Smad3 and NF-κB) downregulate Klotho and PGC-1α expression, whereas PGC-1α expression is promoted by histone crotonylation. A comprehensive understanding of these mediators might contribute to the establishment of novel therapeutic approaches towards CKD prevention and progression [42]. The mechanism of regulation of cell proliferation is worth discussing here. If kidney injury is minor and baseline functionality is normal, the process of repair can be adaptive. However, when the kidney damage is more severe, to a diseased kidney, or repeated, the repair may be maladaptive, and fibrosis may develop due to epithelial cell cycle arrest. Cells going through cell division occupy an extended time span in the G2/M phase, in the ineffective repair process after a renal insult [43]. Cellular senescence is vital in the pathogenicity of CKD. Kidney injury can cause DNA damage, which may lead to senescence. Persistent exposure to reactive oxygen species and repeated proliferation can lead to telomere shortening and senescence [44].

Many genes have been identified in the regulation of signal transduction. These genes are controlled by all listed miRNAs. This emphasizes their involvement in predominantly disease-related processes. Activation of transforming growth factor (TGF)-β is one of the underlying events for the development of CKD, owing to its involvement in the regulation of cell proliferation, fibrogenesis, apoptosis and hypertrophy [45]. This suggests that multiple mechanistic roles are played to determine the outcome of TGF-β signaling. ZBTB7A may serve as a transcription factor when combined with the promoter of *KLF10*, particularly when renal tubular cell growth is reduced. By looking at publicly available transcriptome data, it has been discovered that ZBTB7A is inversely correlated to serum creatinine and significantly decreased in dysfunctional kidneys compared to healthy kidneys. Noteworthy is that transcription factor ZBTB7A positively regulated the proliferative inhibiting impact of *KLF10* in acute kidney injury (AKI) [46].

Xu et al. [47] reported that *SERPINB9* is upregulated in AKI. It is projected that the overexpression of these genes will activate pathways implicated in the production of interleukin 1, 6 and 12, cell death and apoptosis regulation [48,49]. miR-577 was less expressed in kidneys of the cecal ligation and puncture (CLP)-induced AKI mice. Targets of miR-577 were further investigated; *GOLPH3* was selected because miR-577 overexpression significantly reduced GOLPH3 levels. Western blotting data revealed a substantial drop in GOLPH3 protein levels when miR-577 was overexpressed. GOLPH3 was confirmed as the downstream target of miR-577. miR-577 contained the binding site for the 3′-UTR of GOLPH3, negatively controlled its expression and further inhibited translation of the protein [50].

Regulation continues through localization and receptor expression, control of TGF-β family-specific SMAD signaling proteins and collaboration of the SMADs with various signaling pathways which extend inside the nucleus [45]. Immuno-histochemical examination of biopsy samples of kidney has stressed the existence of multiple Wnt proteins common in CKDs [49]. One of the key Wnt ligands that has an established role in the pathogenesis of CKD is Wnt1 [50]. Recently, a study by Kiewisz et al. [51] measured the expression of *Wnt4* in a number of diseases of glomerulus at different degrees of CKD. The absence of *Wnt4* mRNA and protein has been observed in fully functional kidneys just before renal damage or inception of kidney diseases. Consecutive CKD steps exhibit higher Wnt4 manifestation. A significant decline was noticed between stages 2 and 3. Additionally, the transcription factor GLIS2 regulates *Wnt4* expression [52]. Calcium (Ca)-modulating pathways are predicted to be regulated by miR-1-3p, miR-206, miR-494 and miR-656, signifying their interaction with CKD. There is a higher mortality risk due to cardiovascular diseases in CKD patients, and the situation also worsens due to the calcification in the vessels. Dysregulation of calcium metabolism is common in CKD patients [53]. At the sites of necrotic or apoptotic cell death, Ca is released in the walls of blood vessels, and this leads to huge localized elevations in extracellular Ca [54]. Ca-modulating pathways are critical for various physiologic processes entailing muscle contraction, neuronal signaling and blood clotting [55].

**Figure 7:**
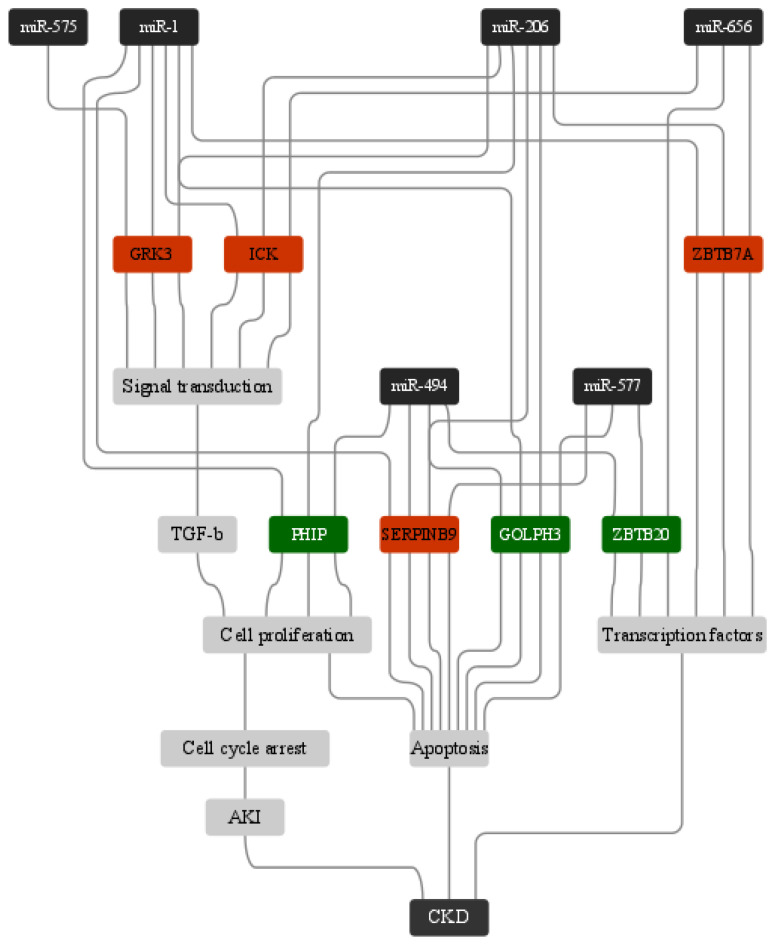
Summary model of the involvement of miRNAs in CKD.

Our research showed that miR-656, miR-577, miR-575, miR-494, miR-206 and miR-1-3p are vital in regulating cellular functions related to the pathogenesis of CKD (Figure 7). Our in silico approach predicted four important miRNAs, and to the best of our knowledge, this is the first study reporting miR-1-3p, miR-575, miR-577 and miR-656 in the development and pathogenesis of CKD. All these predicted miRNAs are significantly linked to the disease condition. However, rigorous in vitro and in vivo experiments are required to validate the diagnostic and therapeutic role of the miRNAs identified in this study.

## 5. Conclusions

The current study elaborated the potential of miRNAs in the pathophysiology of CKD by computational analyses. Several miRNAs were evaluated, and only miR-1-3p, miR-206, miR-494 and miR-577 showed an optimistic future, as they exhibited a strong interaction with CKD. These miRNAs are connected to the expression of certain genes that are involved in important biological processes and key pathways in CKD. Dysregulations of these genes lead to malfunctioning of significant pathways, consequently leading to disease and increased disease severity. Hence, these miRNAs can be acknowledged as potent biomarkers for the detection of CKD and its therapeutic options.

## Figures and Tables

**Table 1:** Number of target DEGs for miRNAs in CKD.

miRNAs	Number of Target DEGs
hsa-miR-1	117
hsa-miR-206	118
hsa-miR-494	204
hsa-miR-575	40
hsa-miR-577	127
hsa-miR-656	149

## Data Availability

Not applicable.

## Supplementary Materials

The following supporting information can be downloaded at: https://www.mdpi.com/article/10.3390/genes14040898/s1, Table S1: Details of miRNA datasets; Table S2: Details of genes datasets.
