# Supplementary material for: Computational Analysis Reveals Distinctive Interaction of miRNAs with Target Genes in the Pathogenesis of Chronic Kidney Disease
Source: Genes (Basel). 2023 Apr 12;14(4):898. doi: 10.3390/genes14040898 (PMC10137451; doi:10.3390/genes14040898)
Supplement: Supplementary file 1 [file genes-14-00898-s001.zip › genes-2272304-supplementary.pdf]

Supplementary Table S1. Details of miRNA datasets.

Sr. No	Accession ID	Gene Expression Platform	Sample Size	No. of Cases	No. of Control	Disease	Reference
**1**	GSE51674	Expression Profiling by Array	16	12	4	Diabetic Nephropathy	Conserva F, Barozzino M, Pesce F, Divella C, Oranger A et al. Urinary miRNA-27b-3p and miRNA-1228-3p correlate with the progression of kidney fibrosis in diabetic nephropathy. Sci. Rep. 2019; 9(1):1-1. https://doi.org/10.1038/s41598-019-47778-1
**2**	GSE89699	Expression Profiling by Array	8	7	1	Chronic Kidney Disease	Vijayaraghavan B, Jeyamohan S, Padmanabhan G, Velangann AJ, Ramanathan K. Circulatory microRNA expression profile for coronary artery calcification in chronic kidney disease patients. Afr. Health Sci. 2021; 21(2):728-34. https://doi.org/10.4314/ahs.v21i2.31
**3**	GSE80247	Expression Profiling by Array	58	21	37	Chronic Kidney Disease	Trevisani F, Ghidini M, Larcher A, Lampis A, Lote H, Papale M et al. MicroRNA 193b-3p as a predictive biomarker of chronic kidney disease in patients undergoing radical nephrectomy for renal cell carcinoma. Br. J. Cancer 2016; 115(11):1343-50. https://doi.org/10.1038/bjc.2016.329

Supplementary Table S2. Details of genes datasets.

Sr. No	Accession ID	Gene Expression Platform	Sample Size	No. of Cases	No. of Control	Disease	Reference
**1**	GSE37171	Expression Profiling by Array	115	75	40	Uremia	Scherer A, Günther OP, Balshaw RF, Hollander Z, Wilson-McManus J, Ng R et al. Alteration of human blood cell tran-scriptome in uremia. BMC Med. Genet. 2013; 6(1):1-3. https://doi.org/10.1186/1755-8794-6-23
**2**	GSE43484	Expression Profiling by Array	6	3	3	Uremia	Al-Chaqmaqchi HA, Moshfegh A, Dadfar E, Paulsson J, Hassan M, Jacobson SH, et al. Activation of Wnt/β-catenin path-way in monocytes derived from chronic kidney disease patients. PLoS One 2013; 8(7): e68937. https://doi.org/10.1371/journal.pone.0068937
**3**	GSE66494	Expression Profiling by Array	61	53	8	Chronic Kidney Disease	Nakagawa S, Nishihara K, Miyata H, Shinke H, Tomita E, Kajiwara M et al. Molecular markers of tubulointerstitial fi-brosis and tubular cell damage in patients with chronic kidney disease. PloS One 2015; 10(8): e0136994. https://doi.org/10.1371/journal.pone.0136994
**4**	GSE142153	Expression Profiling by Array	40	30	10	End Stage Renal Disease	Sur S, Nguyen M, Boada P, Sigdel TK, Sollinger H, Sarwal MM. FcER1: A Novel Molecule Implicated in the Progression of Human Diabetic Kidney Disease. Front. Immunol. 2021; 12. https://doi.org/10.3389/fimmu.2021.769972
